# Amelioration of hepatic steatosis is associated with modulation of gut microbiota and suppression of hepatic miR-34a in *Gynostemma pentaphylla* (Thunb.) Makino treated mice

**DOI:** 10.1186/s12986-018-0323-6

**Published:** 2018-12-05

**Authors:** Ning Jia, Xiaoyan Lin, Shizhan Ma, Shujian Ge, Shumin Mu, Chongbo Yang, Shulong Shi, Ling Gao, Jin Xu, Tao Bo, Jiajun Zhao

**Affiliations:** 1 Shandong University of Traditional Chinese Medicine, Jinan, 250355 China; 2 Department of Endocrinology, Shandong Provincial Hospital affiliated to Shandong University, Jinan, 250021 China; 3 Shandong Provincial Key Laboratory of Institute of Endocrinology and Lipid Metabolism, Jinan, 250021 China; 4 Institute of Endocrinology and Metabolism, Shandong Academy of Clinical Medicine, Jinan, 250021 China; 5 Scientific Center, Shandong Provincial Hospital affiliated to Shandong University, 324, Jing 5 Rd, Jinan, 250021 China; 6 Department of Pathology, Shandong Provincial Hospital affiliated to Shandong University, 324, Jing 5 Rd, Jinan, 250021 China; 7 Department of Scientific Research, Shandong Provincial Hospital affiliated to Shandong University, 324, Jing 5 Rd, Jinan, 250021 China; 8 Department of Endocrinology, Affiliated Hospital of Shandong University of Traditional Chinese Medicine, Jinan, 250014 China

**Keywords:** Non-alcoholic fatty liver disease, *Gynostemma pentaphylla* (Thunb.) Makino, Gut microbiota, miR-34a, Lipid metabolism

## Abstract

**Background:**

Non-alcoholic fatty liver disease (NAFLD) is a chronic and progressive liver disease with an increased risk of morbidity and mortality. However, so far no specific pharmacotherapy has been approved. *Gynostemma pentaphylla* (Thunb.) Makino (GP) is a traditional Chinese medicine that is widely used against hyperlipemia as well as hyperglycemia. This study aims to evaluate the effect of GP on NAFLD and explore the possible mechanism.

**Methods:**

High-fat-diet induced NAFLD mice model were orally administrated with GP at dose of 11.7 g/kg or equivalent volume of distilled water once a day for 16 weeks. Body weight, food intake and energy expenditure were assessed to evaluate the general condition of mice. The triglycerides, total cholesterol content in the liver and liver histopathology, serum lipid profile and serum insulin level, fecal microbiome, hepatic microRNAs and relative target genes were analyzed.

**Results:**

Mice in GP treatment group displayed improved hepatic triglycerides content with lower lipid droplet in hepatocyte and NAFLD activity score. Besides, GP treatment altered the composition of gut microbiota and the relative abundance of some of the key components that are implicated in metabolic disorders, especially phylum *Firmicutes* (*Eubacterium, Blautia, Clostridium and Lactobacillus*). Several hepatic microRNAs were downregulated by GP treatment such as miR-130a, miR-34a, miR-29a, miR-199a, among which the expression miR-34a was altered by more than four-fold compared to that of HFD group (3:14). The correlation analysis showed that miR-34a was strongly related to the change of gut microbiota especially phylum *Firmicut*es (*R* = 0.796)*.* Additionally, the target genes of miR-34a (HNF4α, PPARα and PPARα) were restored by GP both in mRNA and protein levels.

**Conclusion:**

Our results suggested that GP modulated the gut microbiota and suppressed hepatic miR-34a, which was associated with the amelioration of hepatic steatosis.

## Background

Non-alcoholic fatty liver disease (NAFLD) is a chronic progressive disease of the liver that occurs in the absence of excessive alcohol consumption, evolving from simple hepatic steatosis, non-alcoholic steatohepatitis (NASH), fibrosis to cirrhosis and hepatocellular carcinoma (HCC). Convincing evidence has shown that the pathophysiological effects of NAFLD is not only confined to the liver but also extend to multiple systems of human body leading to metabolic comorbidities, cardiovascular disease (CVD) and chronic kidney disease (CKD) [1, 2]. With a global prevalence of 25.24% (22.10–28.65), NAFLD has become a major public health problem [3]. However, so far no pharmacological treatment of this disease is approved [4, 5]. Therefore, it is urgent to find an effective and low-toxicity therapeutic agent against NAFLD.

However, the mechanism of NAFLD is still incompletely understood. Recently, the gut microbiota, a vast microbial community settling in their host’s intestinal tract [6], has emerged as a critical factor in the progression of metabolic disorders especially in NAFLD, as individuals with NAFLD showed significantly different abundance of gut microbiota compared with healthy ones [7, 8] and germ-free (GF) mice that born without exposure to any live microbiota exhibited increased hepatic lipid storage when clonized with a specific microbial profile [9, 10]. Several factors mediate the impact of gut microbiota on host’s energy metabolism such as microbial metabolites, gut permeability and microRNAs (miRNAs), which could be relevant to the initiation and progression of NAFLD [11, 12]. miRNAs are endogenous short non-coding RNAs that act as an crucial regulator in the basic cellular progress through suppressing the post-transcriptional course of target genes [13]. In hepatic cells, gut microbiota-derived metabolites could regulate miRNAs to participate metabolic activity [14] which is recently thought to be a critical link between gut microbiota and liver disease [15, 16]. Notably, dysfunction of specific miRNAs has also been found in the whole progression of NAFLD, suggesting that the gut microbiota-miRNA pathway might be an approach for the therapy of NAFLD.

Traditional Chinese medicine, which has been practiced for centuries in China, has drawn growing attention due to its high efficacy and low side effect on the prevention and treatment of the metabolic disorders especially NAFLD [17, 18]. *Gynostemma pentaphylla* (Thunb.) Makino (GP), a trailing plant that belongs to cucurbitaceae family, has been widely used either alone or as a principal component in herbal formulas for prevention of hyperlipemia as well as hyperglycemia in folks of China and other Asian countries (Korean, Japan etc.) [19]. Pharmacological studies revealed that GP possessed various bioactivities including antioxidation [20], anti-inflammatory [21], hypoglycemia [22, 23], lipid-lowering [24], as well as hepatoprotective effects [25]. Meanwhile, no toxicity were reported at conventional dosage of GP [26]. Clinical evidence suggested that GP decoction combined with diet therapy was more effective than diet therapy for patients with NAFLD [27]. However, no evidence was elucidated about the therapeutic effect GP used alone on NAFLD. Moreover, many herbal medicines were reported to take effect through modulating gut microbiota [28, 29]. While, whether the potential mechanism of GP on NAFLD was through gut microbiota is not clear.

In this study, we evaluated the role of GP in the treatment of NAFLD in vivo and found that GP was effective in reducing the hepatic steatosis and protecting hepatocytes, likely by modulation of gut microbiota and suppression of miR-34a.

## Materials and methods

### Preparation of herbal medicine

The dry aerial parts of GP, authenticated by herbal taxonomist, were obtained from Affiliated Hospital of Shandong University of traditional Chinese Medicine (purchased from Bozhou chengyuan traditional Chinese medicine yinpian CO. LTD, 20161101, Shanxi, China). For extraction, every 100 g of dry herb GP was immersed in 1 L of distilled water for 4 h. Then, the water containing soaked GP was boiled for 40 min. Afterwards, the liquid was poured out and collected while another 1 L of water was added to the residue solids and boiled for another 40 min. Liquid produced from these two boiling was pooled, and concentrated to 1.25 g crude drugs/ml by boiling and then stored at − 80 °C until used. The solution of GP was freshly prepared by dissolving in distilled water before used. Daily dosage of GP used here is chosen based on the Pharmacopoeia of the People’s Republic of China, conversation formula between human per 70 kg and mice per 20 g (1:0.0026) and our preliminary experiments.

### Animal experiments

**Fig. 1:**
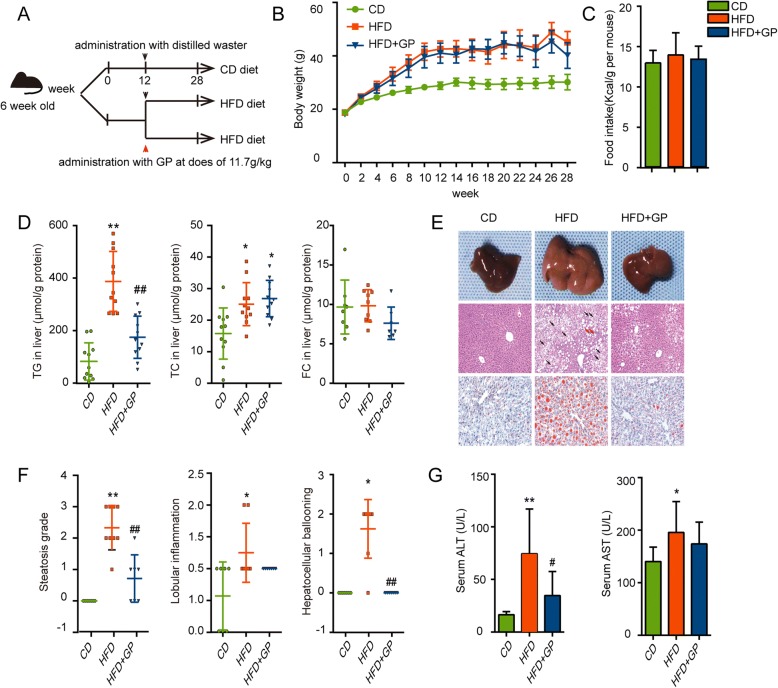
The effect of GP on body weight, food intake, lipid profiles in liver, serum ALT and serum AST. **a** Strategical abstract of the experimental processes. **b** Body weight per mouse during the experiment. **c** Food intake during the treatment of GP. **d** Triglycerides content, total cholesterol content and free cholesterol in liver which were both corrected by the corresponding protein value (*n* = 10–11 per group). **e** Gross morphology of liver tissue selected randomly from four groups and histological analysis of the liver tissue (HE staining × 100 and Oil red O staining × 200). Representative images were shown. Black arrows indicated hepatocellular ballooning and red arrows indicated lobular inflammatory foci. **f** NAFLD activity score, a histological scoring system for NAFLD, was examined, including steatosis grade, lobular inflammation and hepatocellular ballooning (*n* = 6–9 per group). **g** Serum ALT and AST were examined at the end of the experiment (*n* = 8–9 per group). Error bars represent the standards deviation. Statistical analyses were done with one-way ANOVA,*P<0.05,**P<0.01 versus CD;^#^*P*<0.05,^##^*P*<0.01 versus HFD

The strategical abstract of the experimental process was shown in Fig. 1 A. Male C57BL/6 J mice (6-week old) were purchased from Beijing Vital River Laboratory Animal Technology Co., Ltd. All experimental mice were housed in sterilized cages under the condition with 12 h light/dark cycles, 50% humidity, controlled temperature (22–24 °C) and given free access to waster. After acclimatization for 1 week (Fig. 1a), mice were fed with chow diet or high fat diet (HFD, 60% kcal fat, D12492, Research Diets, New Brunswick, NJ, USA) for 12 weeks [30]. Next, mice fed with HFD were divided into GP treatment group (HFD + GP) and model group (HFD), which were respectively orally administrated with GP at a dosage of 11.7 g/kg (12 ml GP extract/kg) or equivalent volume of distilled water once a day for another 16 weeks. Meanwhile, Mice with Chow diet (CD) were orally administrated with the same volume of distilled water like those in HFD group. Body weight and food intake were tested weekly.

### Tissue and serum sample acquisition

After all mice were fasted for 12 h and anesthetized with 1% pentobarbital sodium at the end of 28 weeks, serum was collected after centrifuging at the speed of 3000 rpm for 10 min and the livers were divided into several parts which were fixed in 4% paraformaldehyde solution or frozen immediately in liquid nitrogen and then stored at − 80 °C for subsequent analyses. All of the experimental procedures were implemented in line with the animal study protocol approved by Research Ethics Committee of Shandong University of Traditional Chinese Medicine (Jinan, China).

### Histological analysis of liver

Liver tissues fixed in 4% paraformaldehyde solution (BOSTER biological technology CO., USA) for 24 h were routinely dehydrated, embedded in paraffin and cut into 5 μm slice for hematoxylin and eosin (HE) staining. Frozen section of liver were prepared with cryostat microtome (Leica CM1950, Germany) and cut into 10 μm slice for Oil Red O staining and counterstained with hematoxylin (Goodbio technology CO., LTD, China) for 3 min. All pathological images were observed using a light microscope from several visual fields per slice (Axiovert 100 M Zeiss, Zeppelinstrasse, Germany) at 100× (HE) or 200× (Oil Red O) magnification for the degree of hepatic steatosis. NAFLD activity score (NAS) analysis was determined by a pathologist who was blind to the grouping situation according to the results of HE staining images.

### Measurement of liver lipid accumulation

Liver homogenate was prepared with liver of the same weight after centrifuged at the speed of 12,000 rpm under 4 °C for 20 min. The hepatic triglyceride content and total cholesterol content were measured according to the kit specification (Applygen Technologies Inc., China) which was corrected by the corresponding protein values.

### Serum ALT, AST, glucose levels and insulin levels

The ALT, AST and glucose levels in serum were measured using an Olympus AU5400 automatic biochemical analyzer (Olympus Co., Ltd., Tokyo, Japan). Serum insulin levels were measured with the Mouse Insulin Elisa Kit (CUSABIO, Wuhan, China) according to the manufacturer’s protocol and Homeostasis model assessment-insulin resistance (HOMA-IR) was calculated as following: HOMA-IR = [Fasting blood glucose (FBG, mmol/L) × Fasting insulin level (FINS, mUI/L) / 22.5].

### Metabolic cage analysis

Metabolic cage analysis was performed according to the protocol of the manufacturer (PhenoMaster, TSE Systems, Germany). After 14 weeks of GP treatment, mice were acclimated in the animal monitor chamber for 24 h. Afterwards, volume of O2 consumption (VO2) and CO2 production (VCO2) were recorded regularly with a controlled flow rate over a 24 h period. Respiratory exchange rate (RER) and Heat production was calculated automatically by the installed software.

### Glucose tolerance and insulin tolerance tests

Glucose tolerance and insulin tolerance tests were performed after 15 weeks of GP treatment. For IPGTT, mice fasted for 16 h were injected with 1 g/kg glucose (20% glucose aqueous solution) into the peritoneal cavity. For IPITT, mice fasted for 5 h were injected with 0.8 U/kg insulin (Lilly France S.A.S.)into the peritoneal cavity. Blood was collected from tail vein and glucose levels were monitored using glucometer (ACCU-CHECK performa, Roche, Germany) before injection and at 15, 30, 60, 120 min after injection (IPGTT) or before injection and at 15, 30, 60, 90 min after injection (IPITT). The area under the curve (AUC) was analyzed using GraphPad Prism and the change ratio (% Change) of glucose in IPITT was calculated as following: (each point blood glucose value - initial blood glucose value) / (initial blood glucose value).

### Animal feces samples collection

During feces collection period, six mice were randomly chosen from each group. Totally 18 mice were picked up and housed individually. Feces were collected once per day for 3 continuous days. Sterilized padding was daily changed. Each individual’s feces from 3 days was mixed and then stored at − 80 °C for use.

### DNA extraction, 16S rDNA amplification and high-throughput sequencing

Total microbial DNA was extracted from feces. The 16S rDNA V3-V4 region was amplified by specific degenerate primers (341F: 5′- ACTCCTACGGGRSGCAGCAG -3′; 806R:5′- GGACTACVVGGGTATCTAATC-3′) with unique barcodes suitable for Hiseq2500 PE250. PCR products were excised and extracted using a gel extraction kit (Axyprep DNA gel extraction kit, Axygen). Thermo Nanodrop 2000, Qubit 4.0 and Agilent Bioanalyzer 2100 system were used to perform quality control of DNA samples and DNA library. The eligible libraries were sequenced on an Illumina Hiseq PE250 platform. 250 bp paired-end reads were generated. High-throughput sequencing was performed using the Illumina Hiseq platform by Realgene (Realgene Bioinformation Technology, Shanghai, China).

Sequences (Clean Data) were filtered by Usearch. Sequences with ≥97% similarity were assigned to the same optimal taxonomic units (OTUs). For Alpha diversity analysis, Shannon curves were generated by Quantitative Insights into Microbial Ecology (QIIME, Version 1.7.0) software package and R project (Version 2.15.3). Column chart of relative abundance was draw based on OTU annotation. For Beta diversity, intergroup differences of Beta-diversity were analyzed by Anosim analysis, a non-parametric test. R project was chosen to calculate Principal Co-ordinates Analysis (PCoA). LEfSe analysis was performed to analyze the representative differential species between groups. For LEfSe analysis, Kruskal-Wallis rank sum test was used to detect the species that with significant abundance differences between groups. Grouped-Wilcoxon rank sum test was used to compare the differences between groups based on the species with significant abundance differences. Linear discriminant analysis (LDA) was used to assess the effect of each component, and finds out each group’s right samples that are significantly affected the group partitioning.

### RNA isolation and quantitative real-time PCR (Q-PCR)

miRNAs from liver were isolated using miRcute miRNA isolation kit (Tiangen Biotech, China), as per the manufacturer’s protocols. Total RNA from liver were isolated using Trizol reagent (Takara, Japan) according to manufacturer’s protocols. The concentration of both miRNA and total RNA were measured by a NanoDrop1000 (NanoDrop, USA). Complimentary DNA was synthesized by using miRcute Plus miRNA First-Strand cDNA Synthesis Kit (Tiangen, China) or PrimeScript™ RT reagent Kit (Takara, Japan) according to manufacturer’s instructions. Q-PCR was performed using miRcute Plus miRNA qPCR Detection Kit (Tiangen, China) or SYBR green mix (Bestar qPCR Mastermix, DBI, Germany) and LighCycler 480 (Roche, Mannheim, Germany) according to the protocols. The primers of miRNA and U6 was purchased from Tiangen Biotech, China. Primer sequences of HNF4α, SIRT1 and PPARα were listed in Table 1. The threshold cycle (Ct) of each gene was normalized to U6 miRNA or β-actin mRNA and the fold change was calculated by 2^-△△Ct^ method [31].

**Table 1:** RNA primer

Gene name	Forward and Reverse primer (5′- 3′)
HNF4α	F: GGATATGGCCGACTACAGCG R: GCACCTTCAGATGGGGACG
SIRT1	F: ATATTCCACGGTGCTGAGGT R: TCCAAATCCAGATCCTCCAG
PPARα	F: AAGGGCTTCTTTCGGCGAAC R:TGACCTTGTTCATGTTGAAGTTCTTCA
β-actin	F: GGCTGTATTCCCCTCCATCG R: CCAGTTGGTAACAATGCCATGT

### Western blotting analysis

Liver tissues were lysed in RIPA lysis buffer with protease inhibitors and phosphatase inhibitors (Bimake, Houston, USA) for total protein according to the manufacturer’s instructions (Shenergy Biocolor Bioscience & technology CO., Shanghai, China). Protein concentration was measured using BCA Protein Quantitative Assay Kit (Shenergy Biocolor Bioscience & technology CO., Shanghai, China). The target proteins were blotted with the following antibodies: anti-HNF4α (41770, Invitrogen, USA), anti-SIRT1 (sc-15404, Santa Cruz Biotechnolosy, USA), anti-PPARα (MAB3890, Merck KGaA, Germany), anti-GAPDH (60004–1, Proteintech, China). The relative target protein levels were quantified by densitometry normalized to GAPDH on the same membrane. The samples in one group from three different mice and three independent experiments were performed.

### Statistical analysis

All data was analyzed by IBM SPSS Statistics 22.0 software. The results were demonstrated as mean ± standard deviation (SD) and the statistical analyses were carried out by two-tailed unpaired Student’s t test for two groups and one-way ANOVA test for multiple groups. Pearson’s correlation analyze was utilized to examine the correlations between the abundance of gut microbiota and hepatic miRNAs, and between hepatic miRNAs and hepatic steatosis. *P*-values < 0.05 was considered that differences were significant.

## Results

### GP ameliorates HFD-induced triglycerides deposition in the liver and hepatocytes damage without affecting body weight

HFD led to faster weight gain at the beginning and higher body weight of the mice at the end of the experiment (Fig. 1b) in comparison with chow diet. The effect of GP treatment on body weight or food caloric intake of the HFD fed mice was not significant (Fig. 1b-c). Compared to the non-treated HFD mice, significant reduction in hepatic TG content was observed in GP treatment mice, while hepatic cholesterol content remained unchanged (Fig. 1d). Consistently, GP treatment significantly reduced lipid droplet and ballooning injury induced by HFD in the liver, as shown by HE and Oil Red O staining (Fig. 1e). NAFLD activity score (NAS) showed that GP treatment ameliorated hepatic steatosis grade, lobular inflammations and hepatocellular ballooning (Fig. 1f). In addition, serum AST and ALT levels, two indicators of the acute or chronic hepatocyte impairment, reduced significantly in HFD + GP group compared with that of HFD group (Fig. 1g). These data suggested that GP could reduce hepatic triglycerides deposition without reducing body weight.

### GP improves glucose tolerance and insulin sensitivity

**Fig. 2:**
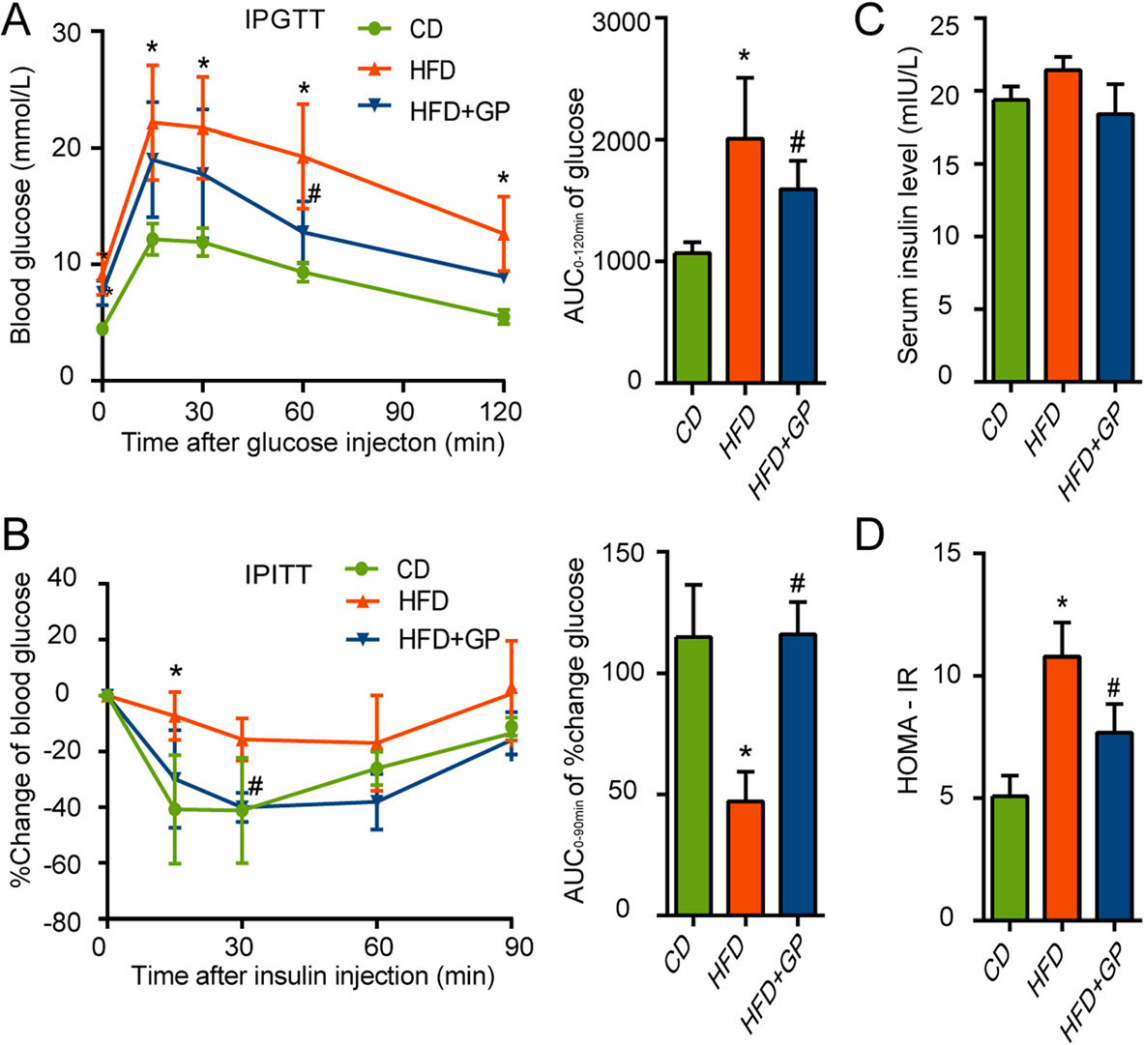
The effect of GP on glucose tolerance and insulin sensitivity. At the end of 15 weeks, IPGTT and IPITT were measured. **a** Effect of GP on the glucose tolerance was determined by intraperitoneal glucose tolerance test (IPGTT) and quantification of the area under the curve (AUC) from the IPGTT (*n* = 4 per group). Effect of GP on the insulin resistance was determined by intraperitoneal insulin tolerance test (IPITT). **b** Percent change of blood glucose during the IPITT was calculated as [(value of certain blood glucose - primary value)/primary value] (*n* = 4–5 per group) and quantification of the area under the curve (AUC) from the % change of glucose. **c** Fasting serum insulin was measured at the end of experiment. **d** Homeostasis model assessment-insulin resistance (HOMA-IR) was calculated as [fasting serum glucose (mmol/L) × fasting serum insulin (mIU/L)/22.5] (*n* = 4–5 per group). Error bars represent the standards deviation Statistical was done by one-way ANOVA, *P<0.05 versus CD,^#^*P*<0.05 versus HFD

Peripheral insulin resistance is frequently thought to be a cardinal accompanying feature of NAFLD and a promoter of the progression of NAFLD [32, 33]. In order to determine if GP could improve insulin resistance, IPGTT and IPITT were measured at the end of 15 weeks after the treatment of GP. Compared with HFD group, GP treatment significantly improved the impaired glucose tolerance and insulin tolerance induced by HFD (Fig. 2a-b). Although no significant difference in serum insulin levels was found between mice in HFD group and HFD + GP group (Fig. 2c), HOMA-IR was significantly reduced in HFD + GP group compared with HFD group (Fig. 2d).

### GP does not affect energy expenditure or serum lipid

**Fig. 3:**
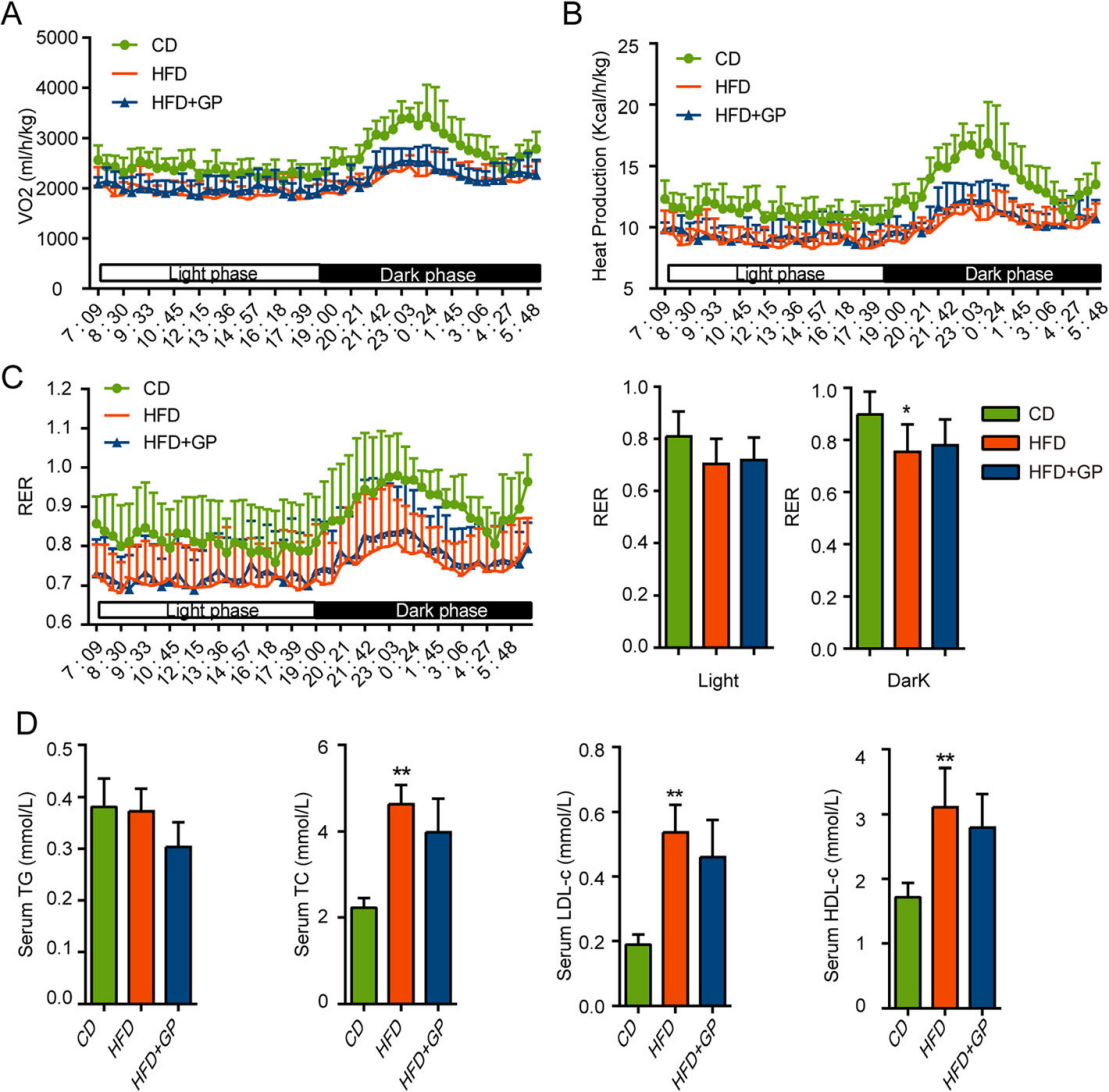
The effect of GP on energy expenditure and serum lipid profiles. At the end of 14 weeks after of GP treatment, energy expenditure were measured including **a** VO2 (volume of oxygen consumed), **b** heat production, **c** RER (respiratory exchange ratio) in the light phase and dark phase by using metabolic cage (*n* = 8 per group). **d** Serum TG, TC, LDL-c and HDL-c levels. Error bars represent the standard deviation. Statistical analyses were done with one-way ANOVA, *P<0.05 versus CD

To evaluate the effect of GP on the whole body energy metabolism, energy expenditure was measured using the metabolic cage analysis. Compared with mice in CD group, HFD mice showed decreased VO2, heat production and RER (Fig. 3a-c) which was consistent with previous studies [34]. While, GP treatment had no effect on these energy metabolism index (Fig. 3a-c). On the other hand, no significant difference in serum lipid profile (TG, TC, LDL-c, HDL-c) was observed between mice in HFD group and HFD + GP group (Fig. 3d).

### GP changes the diversity and abundance of gut microbiota

**Fig. 4:**
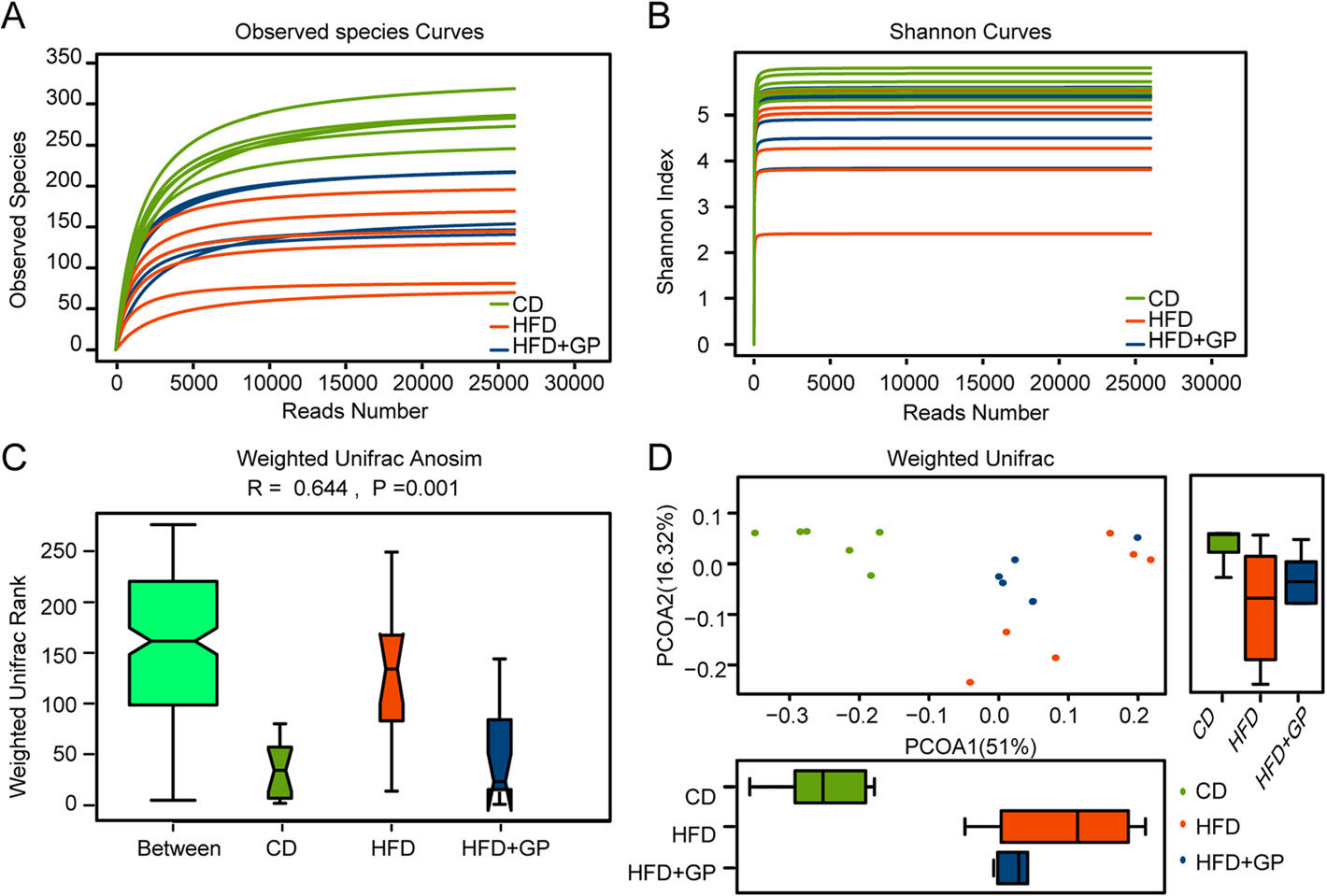
The effect of GP on diversity and abundance of gut microbiota. **a** Observed species analysis. Curves were generated by setting the reads number as X axis, and number of observed OTUs as Y axis. **b** Shannon index analysis. Curves were generated by setting the reads number as X axis, and the Shannon index value as Y axis. Shannon index value indicated the diversity of the samples. **c** Weighted unifrac anosim analysis. Higher weight unifrac rank in between group indicated that the discrepancy among groups was greater than that within the group. R value is between − 1 and 1, *R* > 0 indicates that meaningful differences exist between groups, *R* < 0 indicates that the differences within the group is greater than that between groups. *P* < 0.05 indicates statistical significance. **d** Weighted unifrac PCoA analysis. PCoA1 and PCoA2 in X and Y axis represented two principle discrepancy components between groups, and the percentage in bracket means contribution value to the discrepancies by the component. Dots represent samples. Samples in same group share same color. (*n* = 6 per group)

As the effect of GP on microbiota has yet not to be clearly disclosed, we examined the profile of gut microbiota using 16S rDNA high-throughout sequencing. For alpha diversity analysis, observed species and Shannon index analysis were performed to evaluate the quality and adequacy of microbiome data. Observed species showed that the curves were plateaued with the increase of reads number, which indicated that enough sequences were obtained to cover the majority of species (Fig. 4a). Consistent to other studies [35], the Shannon index of HFD group were lower than that of CD group. In contrast, GP treatment significantly increased the Shannon index (Fig. 4b), which means that the reduction in the diversity of gut microbiota caused by HFD was restored by GP treatment. In order to quantitatively evaluate the differences between groups in more detail, beta-diversity analysis were performed using Unifrac Anosim analysis (Fig. 4c). Unifrac Anosim analysis is one non-parametric test that is used to test whether the differences in sample-structure between groups are significantly greater than that within groups. The R value is calculated and can be used to determine whether the grouping is meaningful. In our result, the sample consistencies of HFD group seemed lower than other two groups, but the average value is obviously higher (Fig. 4c). GP treatment significantly reduced the weighted unifrac rank value, which meant that the structure of HFD + GP group was much closer to CD group than to HFD group. *R* > 0 (0.644) indicated the grouping was meaningful, with statistical significance (*P* = 0.001). Besides sample structure analysis, Principal Coordinates Analysis (PCoA) analysis was used to demonstrate the differences in species diversity between groups (Fig. 4d). PCoA was developed on samples’ distance matrixes, which were generated based on their group-species phylogenic and evolutionary relationships. It can estimate the main discrepancies between groups using the distance of sample-dots. As shown in Fig. 4d, while HFD group and CD group was clearly separated, GP treatment significantly attenuated the effect of HFD and HFD + GP group displayed a shorter distance to CD group in PCoA2 axis. These results indicated that the sample diversity and evolutionary relationships were closer to CD group due to GP treatment under HFD condition.

### GP changes the relative abundance of some metabolic representative species

**Fig. 5:**
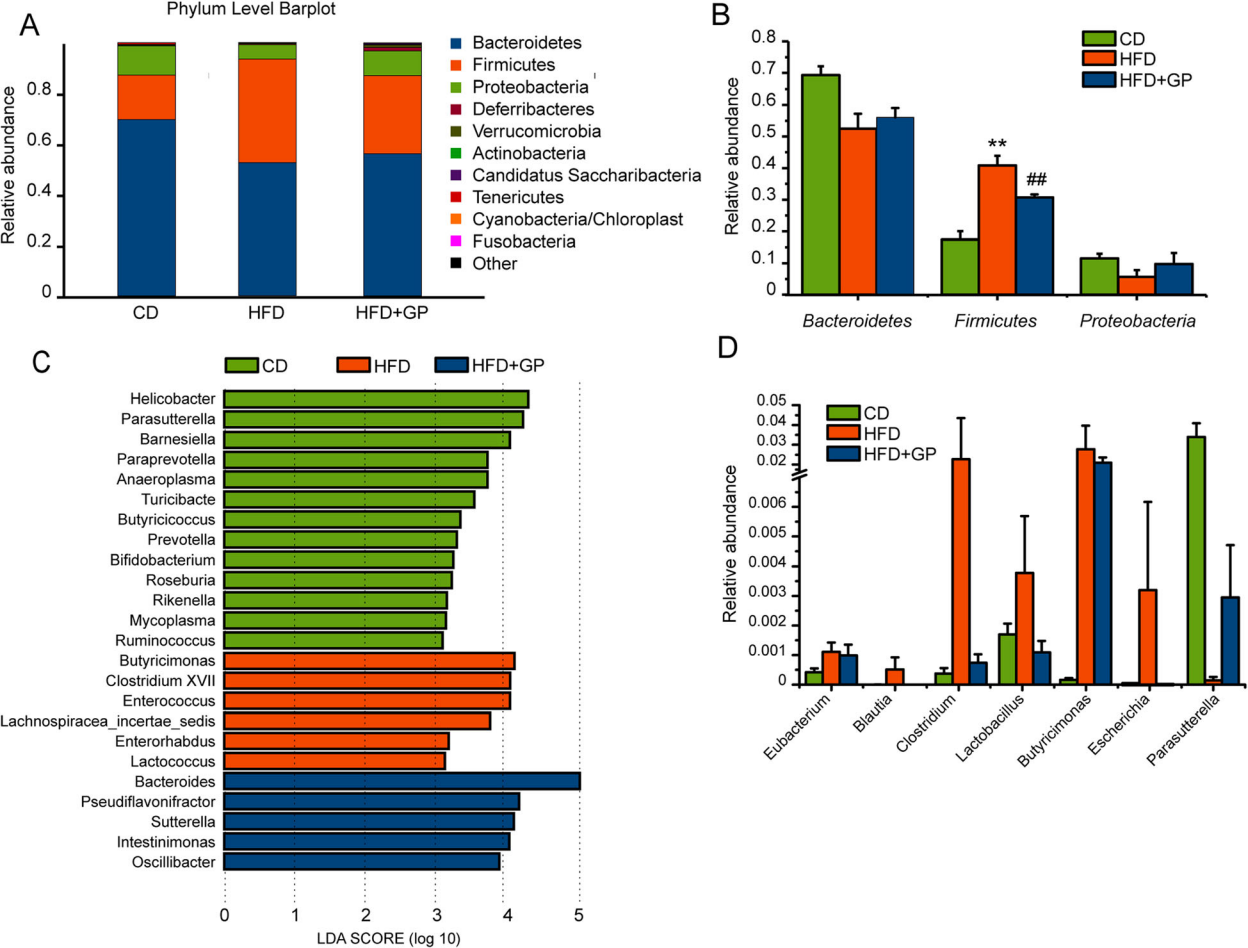
The effect of GP on the relative abundance of some metabolic representative species. **a**-**b** Relative abundance analysis in phylum level. **c** LEfSe analysis of the discrepancies between groups. Gut microbiota was classified in genus level, and the species listed in the left panel was the most representative genera in each group. The log 10 value of LDA scores was set as X axis. **d** Relative abundance analysis in genus level. (*n* = 6 per group)

In phylum levels, significant difference of abundance was found between HFD group and HFD + GP group (Fig. 5a). Specifically, the gut microbiota belonged predominantly to three bacterial phyla: *Bacteroidetes*, *Firmicutes* and *Proteobacteria* (Fig. 5a), among which *Firmicutes* was significantly reduced by GP treatment compared with HFD group (Fig. 5b). To analyze the characteristic species specific within each groups, LEfSe analysis was performed based on the discrepancies between groups. LEfSe uses linear discriminant analysis (LDA) to estimate the effect of abundances of each component, and finds out each group’s right samples that are significantly affected the group partitioning. In the light of LDA results (Fig. 5c), we measured the relative abundances of key components involved in metabolism in each group and the species with positive results were shown in Fig. 5d. In line with previous studies [36, 37], some representative species from phylum *Firmicutes* including genus *Eubacterium*, *Blautia*, *Clostridium* and *Lactobacillus*, increased in HFD group compared with CD group (Fig. 5d). Interestingly, GP treatment could effectively reduce the abundance of these species. Moreover, *Escherichia*, which belongs to phylum *Proteobacteria*, showed the same tendency with those OTUs in *Firmicutes*. Decreased *Parasutterella* (from phylum *Proteobacteria*) induced by HFD was restored in HFD + GP group. These results demonstrated that GP treatment may, directly or indirectly, reverse the effect of HFD on the OTU abundances.

### GP decreases hepatic miR-34a and increased the expression of it related target genes

**Fig. 6:**
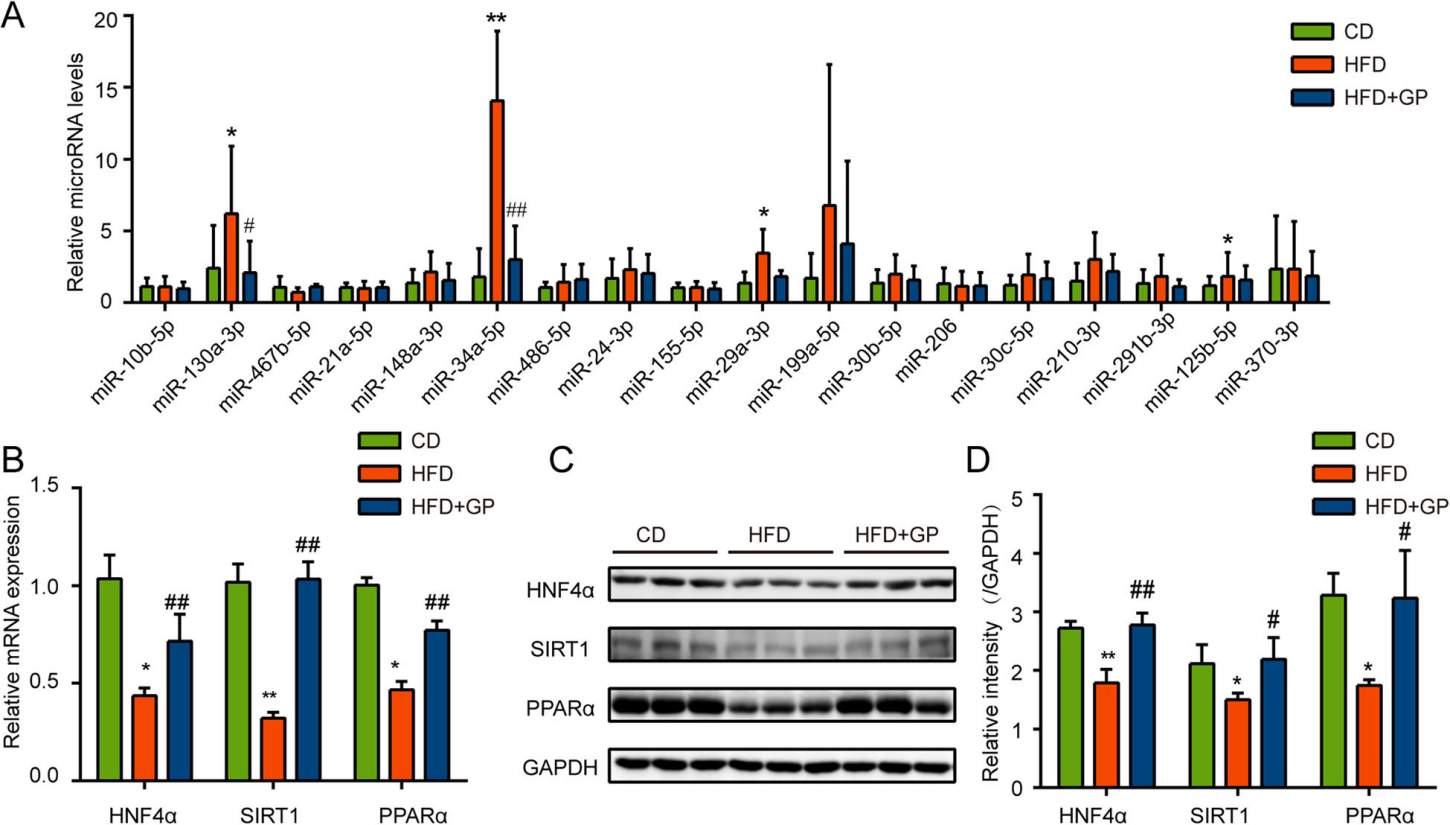
The effect of GP on hepatic miRNAs and relative target genes. **a** Effect of GP on hepatic microRNAs related to the development of NAFLD were analyzed by q-PCR (*n* = 5–7 per group). Hepatic target gene levels triggered by miR34a were determined by q-PCR (**b**) and western-blot (**c**-**d**). Error bars represent the standards deviation. Statistical analyses were done with two-sided Student’s t-test. *P<0.05,**P<0.01 versus CD; ^#^*P*<0.05, ^##^*P*<0.01 versus HFD

Multiple factors were implicated in the role of gut microbiota on metabolic disorders such as endotoxin, altered gut permeability and the regulation of miRNAs. As gut microbial products were reported to participate liver diseases through modulating hepatic miRNAs recently [11, 14], a panel of miRNAs in liver that involved in the development of NAFLD was further analyzed by qPCR (Fig. 6a). While a series of miRNAs were significantly increased by HFD (miR-130a, miR-34a, miR-29a, miR-199a, miR-125b), the expression of miR-130a, miR-34a, miR-29a, miR-199a were decreased by GP treatment (Fig. 6a). To examine whether these changes of hepatic miRNAs was associated with the alteration of gut microbiota, the correlation of hepatic miRNAs and gut microbiota were analyzed (Table 2). The abundance of phylum *Firmicutes* were significantly positively correlated with miR-130a (*R* = 0.499), miR-34a (*R* = 0.796) and miR-29a (*R* = 0.524), among which the intensity of correlation appears between miR-34a and *Firmicutes* was the strongest. Besides, the abundance of phylum *Bacteroidetes* and *Proteobacteria* as well as genus *Parasutterella* showed strong negative correlation with miR-34a. Moreover, the hepatic TG levels and steatosis grade were both positively correlated with miR-34a (Table 3). Based on these results, miR-34a might participate in the regulation of GP on hepatic lipid metabolism.

**Table 2:** Correlation between hepatic miRNAs and gut microbiota

miRNAs	Phylum			Genus						
	*Bacteroidetes*	*Firmicutes*	*Proteobacteria*	*Eubacterium*	*Blautia*	*Clostridium*	*Lactobacillus*	*Butyricimonas*	*Escherichia*	*Parasutterella*
miR-130a-3p	−0.378	0.499^*^	−0.440	0.249	0.001	0.053	0.062	0.087	−0.015	−0.376
miR-34a-5p	−0.613^*^	0.796^**^	−0.586^*^	0.359	0.322	0.279	0.530^*^	0.467	0.286	−0.547^*^
miR-29a-3p	−0.368	0.524^*^	−0.420	0.158	0.183	0.217	0.213	0.232	0.137	−0.466
miR-199a-5p	−0.709^**^	0.340	−0.0167	−0.032	− 0.155	−0.130	− 0.092	−0.126	0.016	−0.320

Values indicate Pearson coefficient of product-moment correlation (*n* = 16). ^*^P<0.05; ^**^P<0.01

**Table 3:** Correlation between hepatic miRNAs and hepatic steatosis

miRNA	Hepatic TG	Steatosis grade
miR-130a-3p	0.458	0.476
miR-34a-5p	0.867^**^	0.862^**^
miR-29a-3p	0.628^*^	0.608^*^
miR-199a-5p	0.313	0.160

Values indicate Pearson coefficient of product-moment correlation (*n* = 16). ^*^P<0.05; ^**^P<0.01

Since miR-34a participates hepatic lipid metabolism by three main target genes including HNF4α, SIRT1 and PPARα [38–40], we further examined the expression of these genes in the liver. In agreement with previous studies, HFD reduced the expression of HNF4α, SIRT1 and PPARα (Fig. 6b-d). Interestingly, such reductions were significantly blocked by GP treatment. These results indicated that miR-34a- related pathway involved in hepatic steatosis were suppressed by GP.

## Discussion

Since hepatic steatosis is the key prerequisite of NAFLD for subsequent events (including NASH, cirrhosis and HCC) and a critical episode in the whole metabolic dysfuction [41], therapeutic strategies against hepatic steatosis has be endowed with a high priority. However, only a limited number of agents have been determined that might be beneficial for this stage with few side effects [5, 42]. In this study, we found that GP ameliorated hepatic steatosis independently of weight loss and raised a novel possible mechanism that the beneficial effect of GP was related to modulation of gut microbiota and suppression of hepatic miR-34a.

As a traditional Chinese medicine, GP contains various bioactive components including saponins, flavone, polysaccharide, amino acids, trace elements and vitamins. Saponins can decrease hepatic steatosis through autophagy modulation [43] and alleviate liver fibrosis induced by carbon tetrachloride (CCl4) in rats [44], which might mediate the beneficial effect of GP on NAFLD, polysaccharides, another main ingredients of GP, has antioxidative, immunomodulatory activities [45] and is able to protect liver from CCl4 injury in mice [46], while flavone was reported to induce cell death in human hepatoma HepG2 cell [47]. Therefore, the components in GP listed above, either acting alone or together, might be responsible for the effect of GP on NAFLD. To be noted, GP as a therapeutic herbal medicine or natural functional food is always used as water decoction or tea drink in clinic. Thus, in this paper the effect of GP on NAFLD was evaluated holistically.

As previously observed [48], NAFLD mice model induced by HFD here developed pronounced hepatic steatosis, insulin resistance and obesity. Obesity plays a direct role in the initiation of NAFLD and sustained weight loss is currently thought to be an effective treatment regimen for NAFLD [5]. However, weight loss is not indispensable for alleviation of hepatic steatosis. For example, mediterranean diet which is reported to be beneficial for hepatic steatosis and insulin resistance, showed no effect on body weight [49]. Actually, not all of obesity patients are followed by NAFLD and on the other hand, a proportion of patients with NAFLD are in the absence of obesity especially in Asia [50]. Although GP failed to affect body weight gain or energy expenditure, it significantly reduced the hepatic steatosis, inflammation, hepatocellular ballooning as well as insulin resistance, which indicated that GP provided beneficial effects on NAFLD independently of weight loss.

Our results, based on community structure and OTU analysis, showed that GP altered the profile of gut microbiota, while decreasing the abundance of phylum *Firmicutes* (e.g. *Eubacterium*, *Blautia*, *Clostridium*, *Lactobacillus*). Similar reduction in phylum *Firmicutes* was found in human with low carbohydrate diet [51]. Most of genus in *Firmicutes* can increase energy extraction from diet which subsequently increase the possibility of lipid accumulation [52, 53]. *Lactobacillus*, in particular, was reported showing a positive association with liver lipid droplets accumulation, through acting as a bile-acid resistant bacteria and contributing to bile acid deconjugation and reduction in fat absorption [54, 55]. Besides, the aberrant abundance of genus *Escherichia* and *Parasutterella* induced by HFD were also alleviated by GP treatment. Several evidences suggested that *Escherichia* derived-ethanol could disrupt gut permeability, ROS generation and liver inflammation, contributing to the development of NAFLD [56, 57]. *Parasutterella* was found decreased in diet induced obesity and metabolic disorders [58], which indicated that it was a protective effect on metabolism. All of these results supported our hypothesis that gut microbiota might mediate the therapeutic effect of GP on NAFLD.

Metabolites derived from gut microbiota are the main intermediary between gut microbiota and their host. In hepatic cells, microbial products were reported to regulate metabolic activity through miRNAs [14] which is recently thought to be a critical link between gut microbiota and host’s disease [15, 16]. For example, incubation of monocytes with *Escherichia coli* lipopolysaccharide (LPS) could upregulate miR-155 and miR-146 to participate the inflammatory response [59]. Another products butyrate was reported to upregulate miR-22 expression and subsequently downregulte the expression of its target genes SIRT-1, resulting in hepatic cell apoptosis [14]. Thus, miRNAs is very likely to be the link between gut microbiota and NAFLD. The miRNAs we scanned were stably expressed in the liver and involved in the course of NAFLD such as hepatic steatosis (miR10b [60], miR-24 [61], miR-29a [62], miR-155 [63], miR-34a [64], miR-125b [65], miR-486 [66], miR-199a [67], miR-30b [68], miR-30c [69], miR467b [70], miR-148a [71]), insulin resistance (miR-125b [72], miR-206 [73], miR-130a [74], miR-291b [75]), inflammatory and hepatic fibrosis (miR-21a [76, 77], miR-199a [78], miR-370 [79], miR-130a [80], miR-34a [81], miR-24 [82]), as well as cirrhosis and HCC (miR-210 [83], miR-10b [84]). We further analyzed the correlation between the alteration of gut microbiota and miRNAs. Interestingly, miR-34a, which was profoundly down-regulated by GP, was the most closely related to the altered microbiota especially phylum *Firmicutes* (genus *Lactobacillus*) induced by GP treatment. miR-34a that was previously deemed as a tumor suppressor, is emerging as a critical miRNA in the progression in NAFLD. It modulates several lipid metabolic pathways in the liver through siting in nucleotide sequences of 3′ untranslated regions (UTRs) of three target genes, including HNF4α, SIRT1 and PPARα, which regulate VLDL secretion, fatty acid synthesis, insulin resistance and fatty acid oxidation to participate the progression of NAFLD [38, 40, 85, 86]. Here, we found that the amelioration of the expression of the three target genes by GP treatment was in consistent with the suppression of miR-34a by GP. Taken together, the effect of GP on gut microbiota was correlated with the modulation of hepatic miR-34a. According to previous studies [7, 87], we thought that gut microbiota-derived metabolites like lipopolysaccharide, ethanol might be responsible to the regulation of miR-34a. However, there are also some studies indicating gut microbiota to be regulated by miRNAs [12, 88]. The possible mechanism may be attributed to the regulation of mRNA profile in bacteria by host miRNAs. Thus, we cannot exclude the possibility that the changes in gut microbiota induced by GP were regulated by hepatic miR-34a. The casual relationship need to be determined by further researches.

## Conclusion

Overall, our study reveals a novel role of GP in alleviating hepatic steatosis and raised the possible mechanism of the beneficial effect of GP through modifying gut microbiota, which was associated with the suppression of hepatic miR-34a. While the results afforded the clues to further research examining the role of GP in human with NAFLD, several additional studies are needed to determine the detailed mechanisms by which GP modify gut microbiota, the host’s hepatic lipid profile, and the activity of miR-34a related pathways.
